# Metabolic Syndrome and Atrial Cardiomyopathy on the Risk of Stroke Mortality in the General Population

**DOI:** 10.1111/anec.70148

**Published:** 2025-12-30

**Authors:** Yaodongqin Xia, Xuan Lu, Minglong Chen, Mingfang Li

**Affiliations:** ^1^ Division of Cardiology The First Affiliated Hospital With Nanjing Medical University Nanjing China; ^2^ Department of Critical Care Medicine Kunshan Hospital of Chinese Medicine Suzhou China; ^3^ Department of Gastroenterology Kunshan Hospital of Chinese Medicine Suzhou China; ^4^ Division of Cardiology The Affiliated Suqian First People's Hospital of Nanjing Medical University Suqian China

**Keywords:** atrial cardiomyopathy, electrocardiography, metabolic syndrome, stroke mortality

## Abstract

**Introduction:**

Metabolic syndrome (MetS) and atrial cardiomyopathy (AtCM) are recognized as risk factors for cardiovascular disease, including stroke. We aimed to determine the combined impact of MetS and AtCM on stroke mortality.

**Methods:**

Participants were selected from the Third National Health and Nutrition Examination (NHANES III) Survey. MetS was defined according to the Adult Treatment Panel III, while AtCM was defined as deep terminal negativity of the P wave in V1 (DTNPV1). Survey‐weighted Firth penalized Cox analysis was performed to determine the adjusted HRs and 95% CIs of stroke mortality by MetS‐AtCM status, including metabolically healthy without AtCM (MHNA; reference), metabolically unhealthy without AtCM (MUNA), metabolically healthy with AtCM (MHA), metabolically unhealthy with AtCM (MUA).

**Results:**

A total of 4315 participants were included in the analysis. Throughout the follow‐up, the rates of stroke mortality increased across the MetS‐AtCM status categories: 1.56, 2.78, 4.68, and 8.24 per 1000 person‐years in MHNA, MUNA, MHA, and MUA groups, respectively. Compared to the MHNA participants, MUA were at a higher risk of stroke mortality (HR = 3.33, 95% CI 1.24–8.94, *p* = 0.018). Stroke mortality showed a non‐significant upward trend in both MUNA (HR = 1.57, 95% CI 0.96–2.59, *p* = 0.074) and MHA (HR = 1.61, 95% CI 0.52–5.00, *p* = 0.401) groups.

**Conclusions:**

Our findings suggest a potential joint association of MetS and AtCM on stroke mortality. Further RCTs are warranted to evaluate the efficacy of anticoagulation in preventing ischemic stroke and reducing stroke mortality among individuals with both MetS and AtCM.

## Graphical abstract

Metabolic syndrome and atrial cardiomyopathy each show a positive but statistically non‐significant association with stroke mortality, but their coexistence confers a synergistically higher risk.

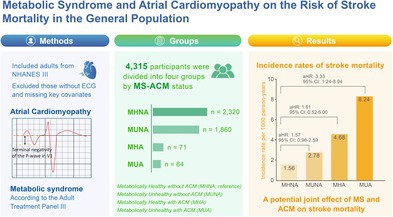

## 1 Introduction

Stroke continues to be a leading cause of mortality worldwide with an annual mortality rate of approximately 5.5 million (Feigin et al. 2022; Sidney et al. 2022). Therefore, the burden of stroke largely lies in its high mortality. In recent years, there has been growing concern about the factors influencing stroke mortality. AF has long been acknowledged as an independent risk factor for stroke, with the highest mortality rate among stroke subtypes according to the TOAST criteria (Kolominsky‐Rabas et al. 2001). Additionally, hypertension, smoking, obstructive sleep apnea, obesity, and underweight are all related to increased risk of fatal stroke as well (Lackland et al. 2014).

Metabolic syndrome (MetS) is a prevalent metabolic disorder characterized by the existence of a cluster of cardiovascular risk factors such as abnormal obesity, hypertension, hyperglycemia, increased triglyceride levels, and low levels of high‐density lipoprotein cholesterol (HDL‐C) (Eckel et al. 2005). MetS has a strong relationship with prevalent stroke and incident stroke (Kurl et al. 2006; Ninomiya et al. 2004). In addition, some components of MetS (low HDL‐C and ≥ 2 components) may predict stroke recurrence (Zhang et al. 2021). Although there is a lack of direct evidence indicating that MetS increases stroke mortality, previous studies have shown an elevated cardiovascular disease mortality, including stroke death, in individuals with MetS (Mottillo et al. 2010).

The concept of atrial cardiomyopathy (AtCM) was proposed in 1972, and it was defined as “any complex of structural, architectural, contractile or electrophysiological changes affecting the atria with the potential to produce clinically relevant manifestations” in 2017 (Goette et al. 2017). As a condition involving structural and functional changes in the atrial myocardium, AtCM has emerged as a significant risk factor for adverse cardiovascular outcomes independent of AF (Ahmad et al. 2020; Kamel et al. 2015; Y. Shen et al. 2022). Prior studies have demonstrated that indicators of AtCM, including abnormal P‐wave parameters (Chen et al. 2022; Maheshwari et al. 2017), elevated N‐terminal brain natriuretic peptide (Ebihara et al. 2020), and abnormal atrial strain function (Maheshwari et al. 2023), are associated with subsequent ischemic stroke independently of AF. The risk of stroke mortality in individuals with AtCM was observed to be 1.76 times higher than that in the general population without AtCM (Ahmad et al. 2020).

However, to date, limited studies have investigated the combined effect of MetS and AtCM on the risk of stroke mortality in the general population. Understanding the interplay between MetS and AtCM in the context of stroke mortality is crucial for the development of effective preventive strategies.

As a non‐invasive tool, electrocardiography (ECG) has been widely used to detect abnormal atrial electrical activity, particularly in defining AtCM across diverse cohorts (Guo et al. 2023; Kamel et al. 2019; Li et al. 2022). Recent evidence indicates that multiple ECG‐derived parameters—such as P‐wave duration, morphology, interatrial block, fibrillatory wave characteristics, and premature atrial contractions—reflect underlying atrial structural and electrical remodeling associated with AtCM (Karakasis, Vlachakis, et al. 2025). Among these indices, the P‐wave terminal force in V1 (PTFV1) has emerged as a key measure of left atrial activation and interatrial conduction (Nakatani et al. 2020). Studies have shown that abnormal PTFV1 is associated with increased risk of AF occurrence (Huang et al. 2020) and ischemic stroke (Guo et al. 2023). Compared to PTFV1, deep terminal negativity of the P‐wave in V1 (DTNPV1) is simpler to use in clinical practice and has consistently shown its strong predictive value for stroke (Tereshchenko et al. 2014). Therefore, we aimed to determine the combined impact of MetS and AtCM defined as abnormal DTNPV1 on stroke mortality in a large, representative sample of the general population.

## 2 Methods

### 2.1 Study Population

The NHANES is a recurring survey assessing the health and nutrition of the U.S. population. Data from NHANES III (1988–1994) were utilized, approved by the NCHS Ethics Review Board with participant consent. It includes demographic, medical history, behavioral data, and laboratory results like cholesterol and triglyceride levels.

**FIGURE 1:**
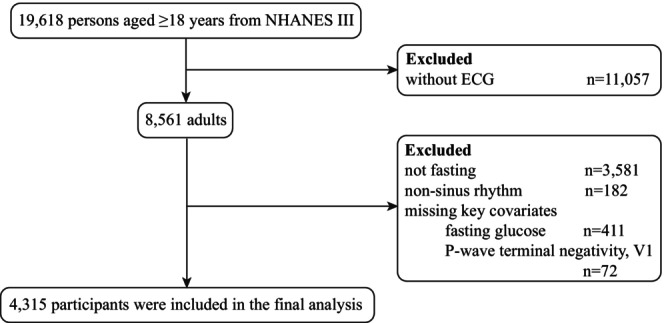
Flow chart.

In this study, 19,618 adults aged ≥ 18 years from NHANES III were considered for enrollment. We excluded participants who did not undergo an electrocardiogram (*n* = 11,057), did not fast for at least 8 h before blood sample collection, had any rhythm other than sinus rhythm, as well as those with missing key covariates for the MetS criteria (*n* = 4246). After all exclusions (*n* = 15,303), 4315 participants were included in the final analysis (Figure 1).

### 2.2 Covariates

Age, sex, race/ethnicity (non‐Hispanic white, non‐Hispanic black, Mexican American, and other), income category (< $20,000/year and ≥ $20,000/year), smoking status (never, former, and current), past medical history (heart failure, stroke, heart attack, hypertension, diabetes mellitus, thyroid disease, cancer, and respiratory disease), and medication history (antiplatelet, anticoagulants, antihypertensive, cardiac glycosides, cholesterol‐lowering, antidiabetic, antiarrhythmic) were self‐reported by the participants. Body mass index (BMI) was calculated based on height and weight measurements, while BP was measured using a mercury sphygmomanometer. Blood samples were collected via venipuncture by a trained phlebotomist. The estimated glomerular filtration rate (eGFR) was calculated by CKD‐EPI (Chronic Kidney Disease Epidemiology Collaboration) equation (Levey et al. 2009). The likelihood of developing cardiovascular disease in every individual was evaluated by calculating the Framingham risk score (D'Agostino et al. 2013).

### 2.3 Electrocardiograph

During the mobile examination visits, trained technicians used a Marquette MAC 12 system (Marquette Medical Systems, Milwaukee, Wisconsin) to acquire resting 12‐lead ECGs. The ECG data were analyzed using a computerized automated process, complemented by visual inspection conducted by a trained technician in a centralized core laboratory. DTNPV1 was diagnosed if the absolute value of the depth of the negative phase of the P wave in lead V1 was > 100 μV when a biphasic P wave was present.

### 2.4 Definition of Atrial Cardiomyopathy and Metabolic Syndrome

In this study, AtCM was defined as DTNPV1. The definition of MetS was based on the National Cholesterol Education Program Adult Treatment Panel III criteria. Participants were considered to have MetS if they met three or more of the following factors: (1) triglyceride ≥ 1.7 mmol/L or on lipid lowering therapy; (2) low HDL‐C < 1.03 mmol/L in men or < 1.3 mmol/L in women; (3) elevated BP (systolic blood pressure > 130 mmHg or diastolic blood pressure > 85 mmHg) or on antihypertensive therapy or self‐reported history of hypertension; (4) fasting glucose > 5.6 mmol/L or self‐reported history of diabetes or on glycemic control therapy; and (5) waist circumference ≥ 102 cm in men or ≥ 88 cm in women. Four categories of AtCM and MetS status were created as follows: metabolically healthy without AtCM (MHNA; reference), metabolically unhealthy without AtCM (MUNA), metabolically healthy with AtCM (MHA), and metabolically unhealthy with AtCM (MUA).

### 2.5 Outcome

The primary outcome of this study was stroke mortality. NHANES III participants were connected to the National Death Index records using a probabilistic matching method. The NHANES III Public‐use Linked Mortality File provides mortality follow‐up data from the date of NHANES III survey participation (1988–1994) through December 31, 2006. The follow‐up duration was defined as the time span from the date of NHANES III participation through the date of death or December 31, 2006, whichever came first. Because the follow‐up period (1988–2006) spanned the transition from ICD‐9 to ICD‐10, causes of death were classified using ICD‐9 codes 430–438 before 1999 and ICD‐10 codes I60–I69 thereafter. In the public‐use mortality file, these codes have been harmonized under UCOD_LEADING = 005, corresponding to “Cerebrovascular diseases (I60–I69)”, which represents the underlying cause of death. Stroke mortality was ascertained from this file.

### 2.6 Statistical Analysis

Baseline characteristics were compared between non‐MetS and MetS groups and further stratified by MetS‐AtCM status (MHNA, MHA, MUNA, and MUA). Continuous variables were reported as mean ± standard deviation (SD) and compared using *t*‐test. Categorical variables were reported as frequencies (percentage) and compared using Chi‐Square test. Bonferroni correction was applied to adjust for multiple comparisons.

Following the NHANES analytic guidelines, survey weights were selected according to the most restrictive variable used in the analysis. Because the definition of MetS required fasting laboratory data, the fasting subsample weight (WTPFSD6) was applied together with strata and primary sampling units (PSUs) to account for the multistage sampling design and ensure representativeness of the fasting subsample.

Weighted Kaplan–Meier curves were generated to estimate stroke‐free survival across MetS–AtCM groups, and differences were compared using the survey‐weighted log‐rank test. To reduce small‐event bias, survey‐weighted Firth penalized Cox models were applied to estimate HR and 95% CI for stroke mortality. Candidate covariates were initially screened using penalized Cox regression with LASSO shrinkage, which included age, sex, race, smoking status, BMI, heart failure, coronary heart disease, prior stroke, antiplatelet, anticoagulant, cardiac glycosides, and antiarrhythmic drugs (Figure S1). Incremental models were specified as follows: (1) Model 1: unadjusted, (2) Model 2: adjusted for age, sex, and race, (3) Model 3: additionally adjusted for smoking, BMI, heart failure, coronary heart disease, and prior stroke, (4) Model 4: further adjusted for antiplatelet, anticoagulant, cardiac glycosides, and antiarrhythmic drugs, (5) Model 5: based on LASSO‐selected variables (age, heart failure, and prior stroke). To explore potential mediation by atrial conduction abnormalities, P‐wave duration, P‐wave axis, and PR interval were incorporated into extended models: (6) Model 6: Model 4 plus P‐wave duration, P‐wave axis, and PR interval, (7) Model 7: Model 5 plus P‐wave duration, P‐wave axis, and PR interval.

To evaluate competing risks, Fine–Gray subdistribution hazard models were fitted using general weighting (sampling weights applied), treating non‐stroke deaths as competing events. The corresponding cumulative incidence function (CIF) curves were plotted to illustrate group‐specific cumulative risks.

Baseline characteristics of excluded versus included participants were compared. Sensitivity analyses included (1) excluding participants with prior stroke and (2) excluding those with prior coronary heart disease or heart failure. Additional sensitivity analyses used a composite ECG‐based definition of AtCM, defined as DTNPV1 > 100 μV, P‐wave duration > 120 ms, or abnormal P‐wave axis (< 0° or > 75°).

All analyses were conducted using SAS version 9.4 and R version 4.2.1; two‐sided *p*‐values < 0.05 were considered statistically significant.

## 3 Results

A total of 4315 participants were included in the final analysis. The average age was 59.5 ± 13.4 years, 51.2% were women, and 48.7% were non‐Hispanic white. The prevalence of MetS and AtCM was 44.6% and 3.1%, respectively. Based on combined MetS–AtCM status, 53.8% were classified as MHNA, 43.1% as MUNA, 1.6% as MHA, and 1.5% as MUA.

The baseline characteristics of participants stratified by their MetS‐AtCM status were summarized in Table 1. Compared with the non‐MetS group, participants with MetS were older, more often female and non‐Hispanic White, and had higher CHA_2_DS_2_‐VASc and Framingham risk scores. They also exhibited a significantly adverse cardiometabolic profile, including higher BMI, waist circumference, blood pressure, fasting glucose, and triglycerides, along with lower HDL‐C and eGFR. Regarding ECG parameters, the MetS group showed a smaller P‐wave axis, longer PR interval, lower positive P‐wave amplitude in leads II and V1, and deeper terminal P‐wave negativity in V1. Moreover, the use of antihypertensive, antidiabetic, and lipid‐lowering medications was higher in the MetS group than in the non‐MetS group. Participants with AtCM were notably older and had higher prevalences of heart failure and coronary heart disease, accompanied by higher CHA_2_DS_2_‐VASc and Framingham risk scores. In comparisons between the non‐AtCM and AtCM groups, the use of cardiac glycosides and antiarrhythmic drugs was also more frequent among those with AtCM.

**TABLE 1:** Clinical characteristics of four metabolic syndrome and atrial cardiomyopathy status categories.

Characteristics, mean ± SD or *n* (%)	Non‐MetS (*n* = 2391)	MetS (*n* = 1924)	*p* ^†^	MHNA (*n* = 2320)	MHA (*n* = 71)	MUNA (*n* = 1860)	MUA (*n* = 64)
Age (years)	57.7 ± 13.6	61.7 ± 12.8	< 0.001	57.5 ± 13.6	65.9 ± 12.9^a^	61.5 ± 12.9	68.7 ± 8.9^b^
Sex (male %)	1236 (51.7)	871 (45.3)	< 0.001	1197 (51.6)	39 (54.9)	843 (45.3)	28 (43.8)
Race (%)			< 0.001				
Non‐Hispanic White (%)	1157 (48.4)	946 (49.2)		1122 (48.4)	35 (49.3)	907 (48.8)	39 (60.9)
Non‐Hispanic Black	609 (25.5)	362 (18.8)		583 (25.1)	26 (36.6)	347 (18.7)	15 (23.4)
Mexican American	514 (21.5)	543 (28.2)		507 (21.9)	7 (9.9)	535 (28.8)	8 (12.5)
Other	111 (4.6)	73 (3.8)		108 (4.7)	3 (4.2)	71 (3.8)	2 (3.1)
Total annual family income > 20,000	1324 (56.4)	884 (47.0)	< 0.001	1293 (56.8)	31 (43.7)	859 (47.2)	25 (41.0)
Weight (kg)	71.5 ± 14.6	82.4 ± 17.2	< 0.001	71.7 ± 14.6	65.6 ± 13.2^a^	82.5 ± 17.2	80.0 ± 16.9
Height (cm)	166.7 ± 9.7	165.1 ± 9.9	< 0.001	166.7 ± 9.7	165.8 ± 9.3	165.1 ± 9.9	164.7 ± 9.5
BMI (kg/m^2^)	25.7 ± 4.5	30.2 ± 5.3	< 0.001	25.7 ± 4.5	23.8 ± 4.0^a^	30.2 ± 5.3	29.2 ± 5.1
Waistline (cm)	91.3 ± 11.4	103.4 ± 11.5	< 0.001	91.4 ± 11.4	87.4 ± 10.6^a^	103.4 ± 11.5	103.0 ± 12.2
Heart rate (bpm)	66.1 ± 10.5	69.2 ± 11.4	< 0.001	66.0 ± 10.4	68.9 ± 13.7	69.0 ± 11.4	72.5 ± 12.1
Systolic blood pressure (mmHg)	126.7 ± 19.1	138.6 ± 19.0	< 0.001	126.4 ± 19.0	137.1 ± 17.2^a^	138.4 ± 18.9	144.7 ± 22.7
Diastolic blood pressure (mmHg)	74.7 ± 10.1	78.0 ± 10.5	< 0.001	74.6 ± 10.0	75.9 ± 11.3	78.1 ± 10.4	77.3 ± 12.4
Heart failure (%)	77 (3.2)	117 (6.1)	< 0.001	69 (3.0)	8 (11.3)^a^	106 (5.7)	11 (17.7)^b^
Coronary heart disease (%)	105 (4.4)	159 (8.3)	< 0.001	95 (4.1)	10 (14.3)^a^	141 (7.7)	18 (28.6)^b^
Hypertension (%)	528 (22.2)	970 (50.6)	< 0.001	501 (21.7)	27 (38.6)^a^	931 (50.2)	39 (60.9)
Diabetes mellitus (%)	77 (3.2)	328 (17.1)	< 0.001	75 (3.2)	2 (2.8)	315 (17.0)	13 (20.3)
Stroke (%)	66 (2.8)	95 (4.9)	< 0.001	62 (2.7)	4 (5.6)	85 (4.6)	10 (15.6)^b^
Thyroid disease (%)	114 (4.8)	99 (5.1)	0.615	108 (4.7)	6 (8.5)	94 (5.1)	5 (7.8)
Cancer (%)	225 (9.4)	208 (10.8)	0.143	214 (9.2)	11 (15.5)	196 (10.5)	12 (18.8)
Respiratory disease (%)	296 (12.6)	268 (14.2)	0.153	283 (12.5)	13 (18.8)	257 (14.1)	11 (18.0)
Smoking (%)			< 0.001				
Current	623 (26.1)	374 (19.4)		597 (25.7)	26 (36.6)	356 (19.1)	18 (28.1)
Former	765 (32.0)	660 (34.3)		743 (32.0)	22 (31.0)	640 (34.4)	20 (31.2)
Never	1003 (41.9)	890 (46.3)		980 (42.2)	23 (32.4)	864 (46.5)	26 (40.6)
Fasting glucose (mmol/L)	5.3 ± 1.1	6.7 ± 2.7	< 0.001	5.3 ± 1.1	5.2 ± 1.0	6.7 ± 2.8	6.4 ± 2.0
Triglycerides (mmol/L)	1.26 ± 0.65	2.37 ± 1.79	< 0.001	1.26 ± 0.66	1.18 ± 0.54	2.37 ± 1.80	2.28 ± 1.47
HDL‐C (mmol/L)	1.46 ± 0.41	1.13 ± 0.33	< 0.001	1.46 ± 0.41	1.59 ± 0.46^a^	1.13 ± 0.33	1.20 ± 0.44
Cholesterol (mmol/L)	5.49 ± 1.06	5.81 ± 1.17	< 0.001	5.49 ± 1.07	5.42 ± 0.87	5.81 ± 1.17	5.90 ± 1.16
Serum creatinine (mmol/L)	97.4 ± 27.9	99.9 ± 41.3	0.019	97.1 ± 27.8	105.3 ± 29.3	99.1 ± 32.5	121.7 ± 142.9^b^
eGFR (mL/(min·1.73 m^2^))	67.9 ± 15.2	64.4 ± 16.7	< 0.001	68.1 ± 15.1	59.8 ± 15.2^a^	64.7 ± 16.7	56.1 ± 14.9^b^
CHA_2_DS_2_‐VASc score	1.4 ± 1.2	2.1 ± 1.5	< 0.001	1.3 ± 1.2	2.1 ± 1.5^a^	2.1 ± 1.5	3.0 ± 1.4^b^
Framingham risk score (%)	18.2 ± 15.2	28.9 ± 17.2	< 0.001	18.0 ± 15.1	25.9 ± 15.1^a^	28.6 ± 17.1	37.7 ± 15.9^b^
P‐wave axis (°)	59.9 ± 24.0	54.7 ± 21.7	< 0.001	59.4 ± 24.0	74.3 ± 17.0^a^	54.4 ± 21.8	62.3 ± 17.1^b^
PR interval (ms)	163.3 ± 27.8	167.3 ± 30.0	< 0.001	162.9 ± 27.6	176.1 ± 32.9^a^	167.1 ± 30.0	173.1 ± 31.2
P‐wave duration (ms)	110.6 ± 15.9	114.0 ± 16.0	< 0.001	110.4 ± 15.9	117.8 ± 16.2^a^	113.9 ± 16.0	119.4 ± 13.3^b^
P‐wave amplitude positive, II (μV)	133.1 ± 48.3	128.1 ± 46.2	0.001	131.8 ± 47.5	176.2 ± 53.5^a^	126.6 ± 45.2	169.5 ± 53.7^b^
P‐wave amplitude positive, V1 (μV)	48.9 ± 29.7	45.5 ± 27.0	< 0.001	49.6 ± 28.8	25.7 ± 45.5^a^	46.1 ± 26.9	29.2 ± 26.6^b^
P‐wave terminal negativity, V1 (μV)	−39.06 ± 27.2	−44.44 ± 26.0	< 0.001	−36.5 ± 22.5	−124.0 ± 30.3^a^	−41.8 ± 21.8	−120.4 ± 22.9^b^
Antiplatelet	40 (1.7)	48 (2.5)	0.073	39 (1.7)	1 (1.4)	44 (2.4)	4 (6.2)
Anticoagulants	16 (0.7)	19 (1.0)	0.323	16 (0.7)	0 (0.0)	18 (1.0)	1 (1.6)
ACE Inhibitors	96 (4.0)	198 (10.3)	< 0.001	84 (3.6)	12 (16.9)^a^	190 (10.2)	8 (12.5)
Beta‐blockers	81 (3.4)	220 (11.4)	< 0.001	78 (3.4)	3 (4.2)	213 (11.5)	7 (10.9)
Calcium Channel Blockers	120 (5.0)	238 (12.4)	< 0.001	115 (5.0)	5 (7.0)	229 (12.3)	9 (14.1)
Diuretics	213 (8.9)	379 (19.7)	< 0.001	202 (8.7)	11 (15.5)	362 (19.5)	17 (26.6)
Other Antihypertensive	34 (1.4)	71 (3.7)	< 0.001	29 (1.2)	5 (7.0)^a^	66 (3.5)	5 (7.8)
Cardiac glycosides	58 (2.4)	80 (4.2)	0.002	52 (2.2)	6 (8.5)^a^	68 (3.7)	12 (18.8)^b^
Cholesterol‐lowering	51 (2.1)	91 (4.7)	< 0.001	47 (2.0)	4 (5.6)	86 (4.6)	5 (7.8)
Antidiabetic	43 (1.8)	218 (11.3)	< 0.001	42 (1.8)	1 (1.4)	211 (11.3)	7 (10.9)
Antiarrhythmic	26 (1.1)	17 (0.9)	0.606	22 (0.9)	4 (5.6)^a^	13 (0.7)	4 (6.2)^b^

Abbreviations: MHA, metabolically healthy with AtCM; MHNA, metabolically healthy without AtCM; MUA, metabolically unhealthy with AtCM; MUNA, metabolically unhealthy without AtCM.

^a^ The difference between MHNA and MHA was statistically significant, with the *p*‐value subjected to Bonferroni correction.

^b^ The difference between MUNA and MUA was statistically significant, with the *p*‐value subjected to Bonferroni correction.

^†^ *p*‐value by *t*‐test for continuous variables and Chi‐square test for categorical variables.

**FIGURE 2:**
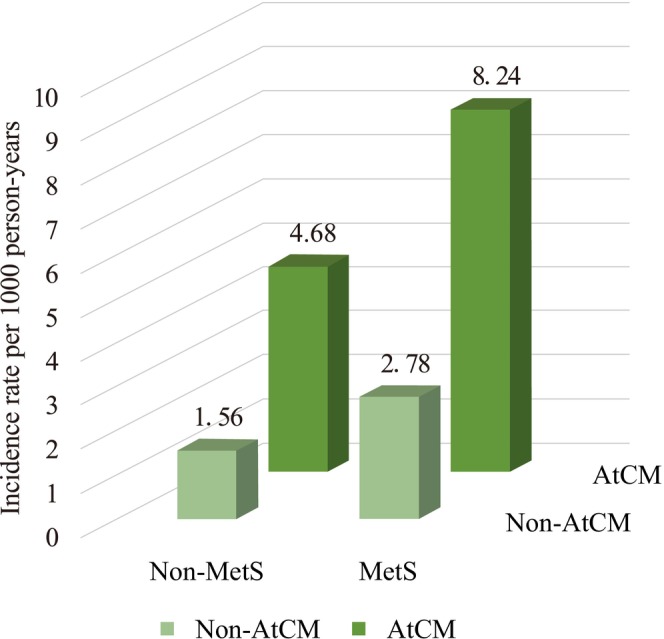
Incidence rates of stroke mortality across metabolic syndrome and atrial cardiomyopathy status categories. AtCM, atrial cardiomyopathy; MetS, metabolic syndrome.

**FIGURE 3:**
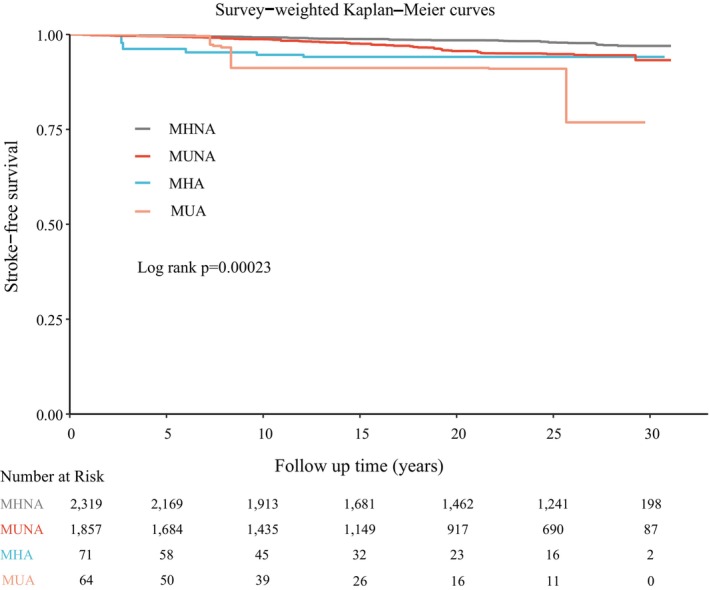
Survey‐weighted Kaplan–Meier curves for stroke‐mortality‐free survival by MetS–AtCM status. MHA, metabolically healthy with AtCM; MHNA, metabolically healthy without AtCM; MUA, metabolically unhealthy with AtCM; MUNA, metabolically unhealthy without AtCM.

At a median follow‐up of 22.7 years (IQR, 11.8–27.6 years), 183 stroke deaths occurred. As shown in Figure 2, the stroke mortality rate increased progressively across the four MetS–ACM status (*p* < 0.001). The incidence rates (per 1000 person‐years) were 1.56 in MHNA, 2.78 in MUNA, 4.68 in MHA, and 8.24 in MUA. Tables S1 and S2 present the absolute risks before and after complex weighting. After weighting, the 10‐year stroke‐mortality‐free survival rates were 99.2%, 98.8%, 94.6%, and 91.2% in the MHNA, MUNA, MHA, and MUA groups, respectively. The weighted Kaplan–Meier curves and corresponding numbers‐at‐risk are displayed in Figure 3.

**FIGURE 4:**
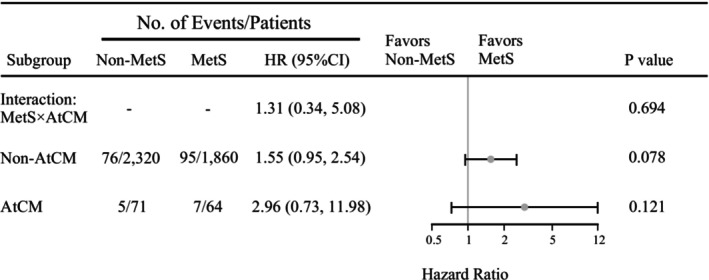
MetS in Different AtCM Subgroups. AtCM, atrial cardiomyopathy; MetS, Metabolic syndrome.

In the survey‐weighted Firth penalized Cox model adjusting for age, heart failure, prior stroke, and P‐wave parameters, both MetS and AtCM were associated with an increased risk of stroke mortality, although the associations did not reach statistical significance. MetS was linked to a 61% higher risk of stroke mortality (adjusted HR = 1.61, 95% CI 0.99–2.62, *p* = 0.053), while AtCM showed an 85% higher risk (adjusted HR = 1.85, 95% CI 0.87–3.93, *p* = 0.109). The interaction term between MetS and AtCM (HR = 1.31, 95% CI 0.34–5.08, *p* = 0.694) also suggested a positive but non‐significant trend (Figure 4).

We further assessed the association of the MetS‐AtCM status with stroke mortality (Table 2). In the fully adjusted survey‐weighted Firth penalized Cox model (Model 7), MUNA and MHA both showed higher risks of stroke mortality compared with MHNA, although these associations did not reach statistical significance (MHA: HR = 1.61, 95% CI 0.52–5.00, *p* = 0.401; MUNA: HR = 1.57, 95% CI 0.96–2.59, *p* = 0.074). MUA had the highest risk of stroke death, with a more than three‐fold increase compared with MHNA (HR = 3.33, 95% CI 1.24–8.94, *p* = 0.018).

**TABLE 2:** Association of metabolic syndrome and atrial cardiomyopathy status categories with stroke mortality.

	MHNA (*n* = 2320)	MHA (*n* = 71)	*p*	MUNA (*n* = 1860)	*p*	MUA (*n* = 64)	*p*
Stroke death (*n* = 183)	76	5		95		7	
Event rate 1000 person‐years (95% CI)	1.56 (reference)	4.68 (1.21–7.42)		2.78 (1.32–2.41)		8.24 (2.44–11.46)	
HR (95% CI), Model 1	1 (reference)	4.22 (1.16–15.33)	0.030	2.25 (1.30–3.89)	0.004	9.45 (3.26–27.40)	< 0.001
HR (95% CI), Model 2	1 (reference)	2.01 (0.73–5.55)	0.174	1.48 (0.92–2.38)	0.103	3.00 (1.03–8.70)	0.044
HR (95% CI), Model 3	1 (reference)	1.95 (0.69–5.55)	0.206	1.62 (0.96–2.74)	0.070	3.38 (1.31–8.70)	0.013
HR (95% CI), Model 4	1 (reference)	2.05 (0.71–5.91)	0.179	1.59 (0.98–2.57)	0.061	3.38 (1.29–8.82)	0.014
HR (95% CI), Model 5	1 (reference)	1.67 (0.53–5.33)	0.376	1.49 (0.91–2.43)	0.111	3.19 (1.15–8.83)	0.027
HR (95% CI), Model 6	1 (reference)	1.86 (0.60–5.76)	0.277	1.62 (1.00–2.62)	0.049	3.44 (1.40–8.48)	0.009
HR (95% CI), Model 7	1 (reference)	1.61 (0.52–5.00)	0.401	1.57 (0.96–2.59)	0.074	3.33 (1.24–8.94)	0.018

*Note:* Model 1 unadjusted. Model 2 adjusted for age, sex and race. Model 3 adjusted for model 2 plus smoking, BMI, heart failure, coronary heart disease and stroke. Model 4 adjusted for model 3 plus antiplatelet, anticoagulant, cardiac glycosides and antiarrhythmic drugs. Model 5 adjusted for age, heart failure and stroke. Model 6 adjusted for model 4 plus P‐wave axis, PR interval, P‐wave duration. Model 7 adjusted for model 5 plus P‐wave axis, PR interval, P‐wave duration.

Abbreviations: MHA, metabolically healthy with AtCM; MHNA, metabolically healthy without AtCM; MUA, metabolically unhealthy with AtCM; MUNA, metabolically unhealthy without AtCM.

**FIGURE 5:**
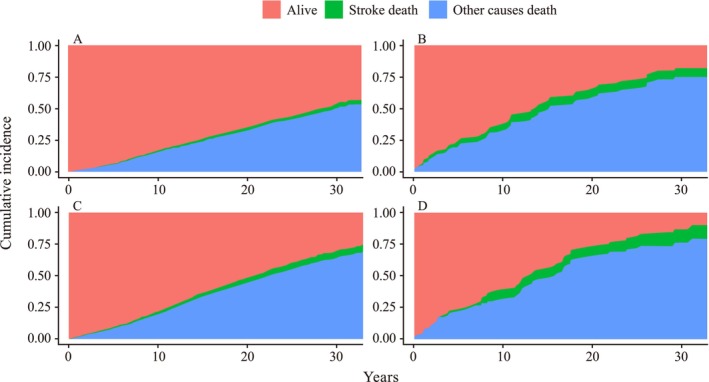
Cumulative incidences for competing risks models of stroke mortality stratified according to metabolic syndrome and atrial cardiomyopathy status categories. (A) Metabolically healthy without AtCM (MHNA); (B) metabolically healthy with AtCM (MHA); (C) metabolically unhealthy without AtCM (MUNA); (D) metabolically unhealthy with AtCM (MUA).

Using the Fine–Gray competing‐risk model (Table S3), the overall pattern of associations was consistent with the primary analysis, although the estimated HRs were lower in magnitude. In the fully adjusted Model 7, the subdistribution hazard ratios (sHRs) were 1.40 (95% CI 1.39–1.41, *p* < 0.001) for MHA, 1.37 (95% CI 1.37–1.38, *p* < 0.001) for MUNA, and 2.24 (95% CI 2.23–2.26, *p* < 0.001) for MUA. The CIF curves (Figure 5) indicated higher cumulative stroke mortality in MUA, consistent with the model estimates.

Compared with the excluded participants, those included in our analytic sample were older, had higher BMI and waist circumference, and showed greater prevalences of hypertension, diabetes, and prior cardiovascular diseases, along with higher fasting glucose and triglyceride levels (Table S4). Sensitivity analyses excluding participants with prior stroke or CHD/HF, and those using a composite ECG‐based definition of AtCM, produced results consistent with the primary analysis, confirming the robustness of our findings (Tables S5–S8).

## 4 Discussion

Our study represents the largest community‐based cohort to date evaluating the combined impact of MetS and AtCM on the risk of stroke mortality in a nationally representative sample of U.S. adults using data from NHANES III. We found that the adjusted odds of stroke mortality were highest in participants who had both MetS and AtCM, as opposed to those with neither condition. Although MetS or AtCM alone showed positive associations with stroke mortality, these did not reach statistical significance after adjustment. Taken together, these findings suggest a joint association between MetS and AtCM, whereby their coexistence confers the greatest risk of stroke‐related death.

The link between MetS and increased risk of stroke mortality revealed in our study may involve a complex interplay of various pathophysiological mechanisms, such as the promotion of atherosclerosis and arteriosclerosis, increased inflammation and oxidative stress, endothelial dysfunction, and abnormalities in blood coagulation and fibrinolysis (Hajhosseiny et al. 2015). The severity of atherosclerosis associated with MetS can make stroke more likely to be fatal by affecting larger brain areas or critical brain regions (van Rooy and Pretorius 2015). MetS is characterized by chronic low‐grade inflammation (Eckel et al. 2005). Elevated inflammatory and thrombogenic biomarkers can upregulate tissue factor, enhance fibrinogen expression, and increase platelet reactivity (Kelly et al. 2021; Lin et al. 2021), which can potentially increase the area of the brain affected and the resulting functional impairment, thereby increasing the risk of stroke mortality (Shi et al. 2019). High glucose‐dependent oxidative stress can lead to impaired Ca^2+^ handling, ion channel dysfunction, cell hypertrophy, and increased apoptosis (Rosa et al. 2013). MetS‐related abnormalities in blood coagulation promote a pro‐thrombotic state, increasing the risk of fatal stroke due to larger or multiple clots (Alessi and Juhan‐Vague 2008).

Also based on the NHANES III, Ahmad et al. found that AtCM could increase the risk of stroke mortality by 76%, with statistical significance (Ahmad et al. 2020). Our study cohort differs from that of Ahmad et al., as we applied additional exclusion criteria, including the exclusion of non‐fasting and missing critical metabolic data. As a result, our final sample size was smaller (4315 vs. 8028 participants). With the smaller analytical sample, the association between AtCM and stroke mortality was not statistically significant (HR = 1.85, 95% CI: 0.87–3.93, *p* = 0.109), but the effect estimate remained directionally consistent with that of Ahmad et al. Our analysis further indicated a consistent and significant trend of increasing stroke mortality risk with worsening metabolic and atrial status, while the interaction between MetS and AtCM remained non‐significant (*p* = 0.694), supporting a joint association.

MetS has been found to be associated with an increased risk of AF (Chamberlain et al. 2010; Watanabe et al. 2008). Furthermore, there is a positive correlation between the number of MetS components and the risk of AF (Ahn et al. 2021), with individuals having all five components of MetS having a risk ratio of 4.4 (95% CI: 3.3–5.9) compared to those without any MetS components (Chamberlain et al. 2010). AtCM could be considered as the underlying substrate of AF. Researchers have delineated the development of AtCM into four stages. The first stage involves the potential onset of AtCM in the atria due to factors such as aging, atrial stretching, inflammation, and oxidative stress. The second stage is reached when there is electrical remodeling, structural remodeling, and autonomic nervous system remodeling in the atria. As the disease progresses, atrial fibrosis and inflammatory reactions may promote the formation of AF and stroke, ultimately leading to the terminal stage of AtCM (M. J. Shen et al. 2019). According to the latest mechanistic review, AtCM arises from a complex interplay of inflammation, oxidative stress, metabolic dysregulation, lipid dicarbonyl stress, epicardial adipose tissue dysfunction, and aging. These processes converge on atrial fibrosis, and on electrical and structural remodeling, forming the mechanistic substrate for atrial fibrillation and thromboembolism (Karakasis, Theofilis, et al. 2025). Taken together, we postulate that the strong combined effect of MetS and AtCM on increased risk of stroke mortality might be attributable to the potential causal effect of MetS and AtCM on AF. However, the relationships between MetS, AtCM, and atrial AF are indeed complex and multifaceted. In addition to the aforementioned fact that both MetS and AtCM contribute to AF, MetS may also serve as a potential pathway to the development of AtCM. In particular, the components of MetS, such as obesity, hypertension, and insulin resistance, have been shown to lead to structural and electrical remodeling of the atria (Hohl et al. 2017; Yilmaz et al. 2015), which in turn increases the risk of AtCM.

Our study has clinical implications. We observed that nearly half of the participants were metabolically unhealthy. In addition, patients with MetS and AtCM had the highest stroke rates, coupled with the highest mortality due to stroke. Interventions aimed at managing MetS components, such as lifestyle modifications (e.g., diet and exercise) and pharmacotherapy (e.g., lipid‐lowering agents, antihypertensive medications, and antidiabetic drugs), may help mitigate the risk of fatal stroke. Moreover, routine screening for AtCM and MetS status, even among those without diagnosed AF, could enable early detection (such as electrocardiography and monitoring the level of glucose, blood pressure, and lipid profiles) and anticoagulant therapy, potentially reducing stroke risk and its mortality.

In our analysis, participants without fasting blood samples or ECG data were excluded, resulting in the removal of nearly three‐quarters of the original NHANES III cohort. This restriction substantially altered the composition of the analytic sample: individuals who remained eligible were older and exhibited higher prevalences of hypertension, diabetes, and other cardiometabolic risk factors, reflecting a metabolically less healthy subset of the U.S. population. Although the use of appropriate fasting examination weights preserves national representativeness for this subsample, the external validity of our findings should be interpreted with caution. Specifically, the associations observed in our study may be more reflective of individuals with greater metabolic burden or those more likely to undergo fasting laboratory assessments, rather than the general adult population. Nevertheless, these analytic restrictions also reduce heterogeneity in metabolic exposure definitions and ensure accurate classification of cardiometabolic status. Thus, while the findings remain robust within the fasting‐eligible population, future studies using more inclusive cohorts with complete metabolic and electrocardiographic data are needed to confirm the broader generalizability of our results.

The strength of our study lies in its large, diverse cohort and the use of NHANES III data, offering a representative snapshot of the U.S. adult population. However, it is not without limitations. First, the observational design precludes causal inference. Second, current consensus emphasizes a multimodality approach to AtCM assessment, integrating echocardiography, cardiac magnetic resonance, and biomarkers to guide evaluation (Karakasis, Vlachakis, et al. 2025; Sade et al. 2025). However, our study defined AtCM solely by ECG parameters. Future studies combining multimodal imaging and biomarker data are needed to enhance the accuracy of AtCM diagnosis and risk stratification. A further limitation is the absence of AF status at baseline and during follow‐up in NHANES III, which leaves the possibility of residual confounding or mediation by incident AF. To address this, we incorporated P‐wave duration, P‐wave axis, and PR interval as proxy markers of atrial electrical remodeling in additional models, and the results remained consistent. Moreover, E‐value analyses indicated that only a relatively strong unmeasured confounder could fully explain the observed associations (Table S9). Although incident AF cannot be entirely excluded as a contributor, it is unlikely to account for the magnitude of the associations observed. Finally, due to current software constraints, we were unable to implement a model that simultaneously integrates Firth penalization, Fine–Gray competing‐risk estimation, and complex survey weighting; therefore, the competing‐risk results based on general weighting should be interpreted as supplementary. Future studies are warranted to explore the mechanisms underlying joint association of MetS and AtCM with increased stroke mortality risk and to investigate potential interventions that could mitigate the elevated stroke mortality risk.

## 5 Conclusion

Our findings suggested a potential joint association of MetS and AtCM on stroke mortality. Further randomized control trials are warranted to evaluate the efficacy of anticoagulation in the prevention of ischemic stroke and stroke mortality among individuals with both MetS and AtCM.

## Author Contributions

Yaodongqin Xia, Minglong Chen, and Mingfang Li contributed to the conception and design of the study. Yaodongqin Xia and Xuan Lu performed the data analysis and drafted the manuscript. Minglong Chen and Mingfang Li critically reviewed and approved the final manuscript. All authors read and approved the final draft.

## Funding

This work was supported by the National Natural Science Foundation of China (grant number 82270329).

## Ethics Statement

This study used de‐identified, publicly available data from NHANES III; no additional ethical approval was needed.

## Conflicts of Interest

The authors declare no conflicts of interest.

## Supporting information


**Appendix S1:** anec70148‐sup‐0001‐AppendixS1.docx.

## Data Availability

The data that support the findings of this study are available in the NHANES III database at the Centers for Disease Control and Prevention (CDC) website: https://wwwn.cdc.gov/nchs/nhanes/nhanes3/default.aspx. These data were derived from the publicly available resource: National Health and Nutrition Examination Survey III (NHANES III), conducted by the National Center for Health Statistics (NCHS) between 1988 and 1994. No reference number is applicable.
