# Supplementary material for: Metabolic Syndrome and Atrial Cardiomyopathy on the Risk of Stroke Mortality in the General Population
Source: Ann Noninvasive Electrocardiol. 2025 Dec 30;31(1):e70148. doi: 10.1111/anec.70148 (PMC12754273; doi:10.1111/anec.70148)
Supplement: Supplementary file 1 — Appendix S1: anec70148‐sup‐0001‐AppendixS1.docx. [file ANEC-31-e70148-s001.docx]

Supplementary Figure 1. Variable selection by LASSO penalized Cox regression.

1. cross-validation for optimal λ and (B) coefficient profiles across log(λ) values


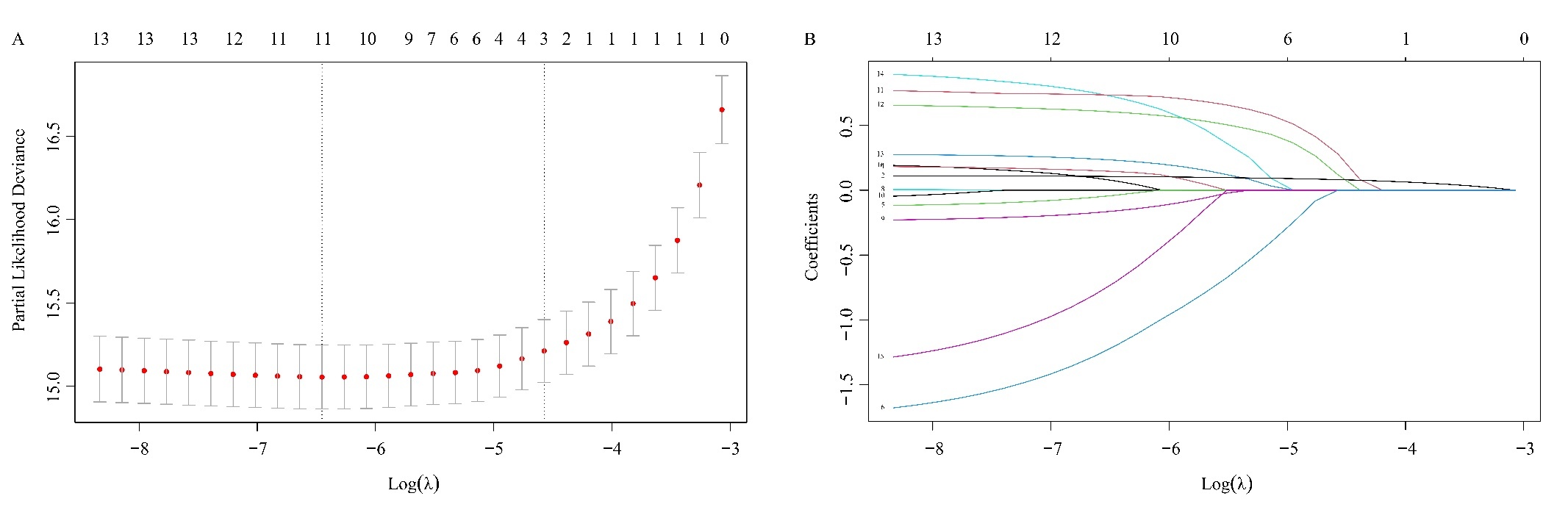


Figure Legends: Candidate covariates included age, sex, race, smoking status, BMI, heart failure, coronary heart disease, prior stroke, and medication use (antiplatelet agents, anticoagulants, cardiac glycosides, and antiarrhythmic drugs). Variables with non-zero coefficients at the optimal λ were selected for inclusion in subsequent multivariable models.

Supplementary Table 1. Absolute risks of stroke mortality (%) estimated without weighting

| Years of follow-up | 5 years | 10 years | 15 years | 20 years | 25 years | 30 years |
| --- | --- | --- | --- | --- | --- | --- |
| MHNA | 0.4%  (0.1–0.6%) | 1.4%  (0.9–1.9%) | 2.2%  (1.5–2.8%) | 2.7%  (2.0–3.5%) | 3.8%  (2.9–4.7%) | 4.9%  (3.7–6.1%) |
| MUNA | 1.1%  (0.6–1.6%) | 2.4%  (1.7–3.1%) | 3.9%  (2.9–4.9%) | 5.6%  (4.3–6.8%) | 7.0%  (5.5–8.4%) | 8.3%  (6.3–10.2%) |
| MHA | 3.0%  (0.0–6.9%) | 6.8%  (0.1–13.1%) | 9.2%  (1.0–16.7%) | 9.2%  (1.0–16.7%) | 9.2%  (1.0–16.7%) | 9.2%  (1.0–16.7%) |
| MUA | 1.9%  (0.0–5.5%) | 10.6%  (1.3–19.0%) | 10.6%  (1.3–19.0%) | 10.6%  (1.3–19.0%) | 16.6%  (1.4–29.4%) | 16.6%  (1.4–29.4%) |

Supplementary Table 2. Absolute risks of stroke mortality (%) estimated with complex survey weighting

| Years of follow-up | 5 years | 10 years | 15 years | 20 years | 25 years | 30 years |
| --- | --- | --- | --- | --- | --- | --- |
| MHNA | 0.2% | 0.8% | 1.2% | 1.5% | 2.1% | 3.0% |
| MUNA | 0.6% | 1.3% | 2.5% | 4.4% | 5.2% | 6.8% |
| MHA | 3.8% | 5.4% | 5.9% | 5.9% | 5.9% | 5.9% |
| MUA | 0.4% | 8.8% | 8.8% | 8.8% | 23.2% | 23.2% |

Note: Metabolically healthy without AtCM (MHNA), metabolically unhealthy without AtCM (MUNA), metabolically healthy with AtCM (MHA), metabolically unhealthy with AtCM (MUA).

Supplementary Table 3. Fine–Gray subdistribution hazard models estimated with general weighting

|  | MHNA | MHA | P | MUNA | P | MUA | P |
| --- | --- | --- | --- | --- | --- | --- | --- |
| Model 1 | - | 2.67  (2.65-2.69) | <.001 | 1.89  (1.88-1.89) | <.001 | 5.00  (4.98-5.03) | <.001 |
| Model 2 | - | 1.43  (1.42-1.44) | <.001 | 1.35  (1.35-1.36) | <.001 | 2.42  (2.41-2.43) | <.001 |
| Model 3 | - | 1.55  (1.53-1.56) | <.001 | 1.48  (1.48-1.48) | <.001 | 2.67  (2.66-2.69) | <.001 |
| Model 4 | - | 1.70  (1.69-1.71) | <.001 | 1.48  (1.48-1.49) | <.001 | 2.87  (2.86-2.89) | <.001 |
| Model 5 | - | 1.46  (1.45-1.47) | <.001 | 1.35  (1.35-1.35) | <.001 | 2.27  (2.26-2.28) | <.001 |
| Model 6 | - | 1.56  (1.55-1.57) | <.001 | 1.47  (1.47-1.48) | <.001 | 2.82  (2.80-2.83) | <.001 |
| Model 7 | - | 1.40  (1.39-1.41) | <.001 | 1.37  (1.37-1.38) | <.001 | 2.24  (2.23-2.26) | <.001 |

Note: Model 1 unadjusted.

Model 2 adjusted for age, sex and race.

Model 3 adjusted for model 2 plus smoking, BMI, heart failure, coronary heart disease and stroke.

Model 4 adjusted for model 3 plus antiplatelet, anticoagulant, cardiac glycosides and antiarrhythmic drugs.

Model5 penalized model adjusted for LASSO-selected predictors (age, heart failure, and prior stroke).

Model6 adjusted for model 4 plus P-wave axis, PR interval, P-wave duration.

Model7 adjusted for model 5 plus P-wave axis, PR interval, P-wave duration.

Metabolically healthy without AtCM (MHNA), metabolically unhealthy without AtCM (MUNA), metabolically healthy with AtCM (MHA), metabolically unhealthy with AtCM (MUA).

Supplementary Table 4. Baseline characteristics of included versus excluded participants

|  | Excluded (n=15303) | Included (n=4315) |
| --- | --- | --- |
| Age, years | 45.06 (21.06) | 59.49 (13.40) |
| Sex (male, %) | 7086 (46.3) | 2107 (48.8) |
| Ethnicity (%) |  |  |
| Mexican American | 4096 (26.8) | 1057 (24.5) |
| Non-Hispanic black | 4374 (28.6) | 971 (22.5) |
| Non-Hispanic white | 6266 (40.9) | 2103 (48.7) |
| Other | 567 (3.7) | 184 (4.3) |
| Weight, kg | 74.05 (18.31) | 76.37 (16.74) |
| Height, cm | 166.49 (9.83) | 165.97 (9.82) |
| BMI, kg/m² | 26.66 (5.96) | 27.67 (5.32) |
| Waist circumference, cm | 91.38 (14.87) | 96.69 (12.93) |
| Heart rate, bpm | 72.42 (11.12) | 67.45 (11.04) |
| Systolic BP, mmHg | 124.73 (20.54) | 132.01 (19.93) |
| Diastolic BP, mmHg | 73.55 (11.28) | 76.17 (10.39) |
| Heart failure (%) | 562 (3.7) | 194 (4.5) |
| Coronary heart disease (%) | 674 (4.5) | 264 (6.1) |
| Hypertension (%) | 3924 (25.9) | 1498 (34.7) |
| Diabetes (%) | 1207 (7.9) | 405 (9.4) |
| Stroke (%) | 488 (3.2) | 161 (3.7) |
| Smoking status (%) |  |  |
| Current | 3948 (25.8) | 997 (23.1) |
| Former | 3366 (22.0) | 1425 (33.0) |
| Never | 7971 (52.1) | 1893 (43.9) |
| Serum glucose, mmol/L | 5.46 (2.09) | 5.92 (2.12) |
| Triglycerides, mmol/L | 1.58 (1.25) | 1.75 (1.40) |
| HDL cholesterol, mmol/L | 1.33 (0.40) | 1.32 (0.41) |
| Total cholesterol, mmol/L | 5.17 (1.16) | 5.63 (1.12) |
| Serum creatinine, µmol/L | 95.48 (33.27) | 98.49 (34.54) |

Supplementary Table 5. Survey-weighted Firth penalized Cox model excluding participants with prior stroke

|  | MHNA | MHA | P | MUNA | P | MUA | P |
| --- | --- | --- | --- | --- | --- | --- | --- |
| Model 1 | - | 3.85(0.95–15.62) | 0.059 | 2.06 (1.17–3.63) | 0.014 | 10.48 (3.48-31.57) | <.001 |
| Model 2 | - | 1.83 (0.60–5.57) | 0.280 | 1.35 (0.83–2.19) | 0.223 | 3.33 (1.08–10.22) | 0.036 |
| Model 3 | - | 1.78 (0.58–5.45) | 0.305 | 1.42 (0.87–2.31) | 0.157 | 3.49 (1.28–9.52) | 0.016 |
| Model 4 | - | 1.83 (0.59–5.68) | 0.287 | 1.44 (0.89–2.33) | 0.131 | 3.36 (1.23–9.22) | 0.020 |
| Model 5 | - | 1.43 (0.40–5.12) | 0.574 | 1.39 (0.85–2.29) | 0.189 | 3.47 (1.22–9.85) | 0.021 |
| Model 6 | - | 1.59 (0.46–5.49) | 0.455 | 1.46 (0.91–2.34) | 0.138 | 3.43 (1.23–9.53) | 0.010 |
| Model 7 | - | 1.38 (0.40–4.80) | 0.607 | 1.44 (0.86–2.42) | 0.165 | 3.65 (1.40–9.47) | 0.009 |

Supplementary Table 6. Survey-weighted Firth penalized Cox model excluding participants with prior CHD/HF

|  | MHNA | MHA | P | MUNA | P | MUA | P |
| --- | --- | --- | --- | --- | --- | --- | --- |
| Model 1 | - | 4.91 (1.30-18.52) | 0.020 | 2.14 (1.20-3.82) | 0.011 | 12.19 (3.74-39.75) | <.001 |
| Model 2 | - | 2.29 (0.83-6.34) | 0.108 | 1.42 (0.87-2.34) | 0.159 | 3.66 (1.12-12.00) | 0.033 |
| Model 3 | - | 2.16 (0.76-6.18) | 0.147 | 1.56 (0.90-2.73) | 0.113 | 3.91 (1.37-11.22) | 0.012 |
| Model 4 | - | 2.22 (0.77-6.39) | 0.138 | 1.53 (0.91-2.58) | 0.110 | 3.92 (1.34-11.45) | 0.014 |
| Model 5 | - | 1.84 (0.55-6.11) | 0.314 | 1.47 (0.89-2.43) | 0.133 | 4.08 (1.32-12.58) | 0.015 |
| Model 6 | - | 2.08 (0.67-6.44) | 0.201 | 1.57 (0.94-2.63) | 0.084 | 4.08 (1.51-11.01) | 0.007 |
| Model 7 | - | 1.83 (0.57-5.91) | 0.303 | 1.54 (0.92-2.57) | 0.096 | 4.34 (1.53-12.29) | 0.007 |

Note: Model 1 unadjusted.

Model 2 adjusted for age, sex and race.

Model 3 adjusted for model 2 plus smoking, BMI, heart failure, coronary heart disease and stroke.

Model 4 adjusted for model 3 plus antiplatelet, anticoagulant, cardiac glycosides and antiarrhythmic drugs.

Model5 penalized model adjusted for LASSO-selected predictors (age, heart failure, and prior stroke).

Model6 adjusted for model 4 plus P-wave axis, PR interval, P-wave duration.

Model7 adjusted for model 5 plus P-wave axis, PR interval, P-wave duration.

Metabolically healthy without AtCM (MHNA), metabolically unhealthy without AtCM (MUNA), metabolically healthy with AtCM (MHA), metabolically unhealthy with AtCM (MUA).

Supplementary Table 7. Stroke mortality events by MetS–AtCM group using a composite ECG-based definition of atrial cardiomyopathy

|  | Survivors | Stroke deaths | Total participants |
| --- | --- | --- | --- |
| MHNA | 1,256 | 29 | 1,285 |
| MUNA | 1,036 | 48 | 1,084 |
| MHA | 1,053 | 52 | 1,105 |
| MUA | 783 | 54 | 837 |

Note: Atrial cardiomyopathy was defined as the presence of any of the following ECG abnormalities: deep terminal negativity of the P wave in V1 (DTNPV1 > 100 μV), P-wave duration > 120 ms, or abnormal P-wave axis (< 0° or > 75°).

Supplementary Table 8. Sensitivity analysis using a composite ECG-based definition of atrial cardiomyopathy (AtCM) showing results consistent with the primary analysis

|  | MHNA | MHA | P | MUNA | P | MUA | P |
| --- | --- | --- | --- | --- | --- | --- | --- |
| Model 1 | - | 3.62 (1.83–7.15) | <0.001 | 4.36 (2.66–7.14) | <0.001 | 5.40 (3.32–8.80) | <0.001 |
| Model 2 | - | 2.36 (1.27–4.39) | 0.008 | 2.94 (1.87–4.63) | <0.001 | 2.19 (1.26–3.79) | 0.006 |
| Model 3 | - | 2.42 (1.27–4.62) | 0.009 | 3.21 (1.89–5.47) | <0.001 | 2.34 (1.28–4.30) | 0.007 |
| Model 4 | - | 2.53 (1.29–4.95) | 0.008 | 3.11 (1.86–5.19) | <0.001 | 2.43 (1.36–4.35) | 0.003 |
| Model 5 | - | 2.37 (1.25–4.49) | 0.009 | 2.96 (1.87–4.70) | <0.001 | 2.20 (1.30–3.71) | 0.004 |

Note: Model 1 unadjusted.

Model 2 adjusted for age, sex and race.

Model 3 adjusted for model 2 plus smoking, BMI, heart failure, coronary heart disease and stroke.

Model 4 adjusted for model 3 plus antiplatelet, anticoagulant, cardiac glycosides and antiarrhythmic drugs.

Model5 penalized model adjusted for LASSO-selected predictors (age, heart failure, and prior stroke).

Metabolically healthy without AtCM (MHNA), metabolically unhealthy without AtCM (MUNA), metabolically healthy with AtCM (MHA), metabolically unhealthy with AtCM (MUA).

Supplementary Table 9. E-values for survey-weighted Firth penalized Cox models of stroke mortality

|  | MHNA | MHA | MUNA | MUA |
| --- | --- | --- | --- | --- |
| Model 1 | - | 7.906 | 3.927 | 18.386 |
| Model 2 | - | 3.434 | 2.323 | 5.449 |
| Model 3 | - | 3.311 | 2.708 | 6.057 |
| Model 4 | - | 3.548 | 2.645 | 6.057 |
| Model 5 | - | 2.964 | 2.340 | 5.718 |
| Model 6 | - | 3.125 | 2.622 | 6.337 |
| Model 7 | - | 2.601 | 2.516 | 6.115 |

Model 1 unadjusted.

Model 2 adjusted for age, sex and race.

Model 3 adjusted for model 2 plus smoking, BMI, HF, coronary heart disease and stroke.

Model 4 adjusted for model 3 plus antiplatelet, anticoagulant, cardiac glycosides and antiarrhythmic drugs.

Model 5 penalized model adjusted for LASSO-selected predictors (age, heart failure, and prior stroke).

Model 6 adjusted for model 4 plus P-wave axis, PR interval, P-wave duration.

Model 7 adjusted for model 5 plus P-wave axis, PR interval, P-wave duration.
